# Inhibition of NADPH oxidase 2 enhances resistance to viral neuroinflammation by facilitating M1-polarization of macrophages at the extraneural tissues

**DOI:** 10.1186/s12974-024-03078-8

**Published:** 2024-05-02

**Authors:** Jin Young Choi, Hee Won Byeon, Seong Ok Park, Erdenebileg Uyangaa, Koanhoi Kim, Seong Kug Eo

**Affiliations:** 1 College of Veterinary Medicine and Bio-Safety Research Institute, Jeonbuk National University, Iksan, 54596 Republic of Korea; 2 Department of Pharmacology, School of Medicine, Pusan National University, Yangsan, 50612 Republic of Korea

**Keywords:** NADPH oxidase 2, Japanese encephalitis, Macrophages, Reactive oxygen species, ROS scavenger

## Abstract

**Background:**

Macrophages play a pivotal role in the regulation of Japanese encephalitis (JE), a severe neuroinflammation in the central nervous system (CNS) following infection with JE virus (JEV). Macrophages are known for their heterogeneity, polarizing into M1 or M2 phenotypes in the context of various immunopathological diseases. A comprehensive understanding of macrophage polarization and its relevance to JE progression holds significant promise for advancing JE control and therapeutic strategies.

**Methods:**

To elucidate the role of NADPH oxidase-derived reactive oxygen species (ROS) in JE progression, we assessed viral load, M1 macrophage accumulation, and cytokine production in WT and NADPH oxidase 2 (NOX2)-deficient mice using murine JE model. Additionally, we employed bone marrow (BM) cell-derived macrophages to delineate ROS-mediated regulation of macrophage polarization by ROS following JEV infection.

**Results:**

NOX2-deficient mice exhibited increased resistance to JE progression rather than heightened susceptibility, driven by the regulation of macrophage polarization. These mice displayed reduced viral loads in peripheral lymphoid tissues and the CNS, along with diminished infiltration of inflammatory cells into the CNS, thereby resulting in attenuated neuroinflammation. Additionally, NOX2-deficient mice exhibited enhanced JEV-specific Th1 CD4 ^+^ and CD8 ^+^ T cell responses and increased accumulation of M1 macrophages producing IL-12p40 and iNOS in peripheral lymphoid and inflamed extraneural tissues. Mechanistic investigations revealed that NOX2-deficient macrophages displayed a more pronounced differentiation into M1 phenotypes in response to JEV infection, thereby leading to the suppression of viral replication. Importantly, the administration of H_2_O_2_ generated by NOX2 was shown to inhibit M1 macrophage polarization. Finally, oral administration of the ROS scavenger, butylated hydroxyanisole (BHA), bolstered resistance to JE progression and reduced viral loads in both extraneural tissues and the CNS, along with facilitated accumulation of M1 macrophages.

**Conclusion:**

In light of our results, it is suggested that ROS generated by NOX2 play a role in undermining the control of JEV replication within peripheral extraneural tissues, primarily by suppressing M1 macrophage polarization. Subsequently, this leads to an augmentation in the viral load invading the CNS, thereby facilitating JE progression. Hence, our findings ultimately underscore the significance of ROS-mediated macrophage polarization in the context of JE progression initiated JEV infection.

## Introduction

Japanese encephalitis (JE) represents the leading malady within the spectrum of viral encephalitis, a severe neurological inflammatory condition manifested by the disruption of blood–brain barrier (BBB) and the infiltration of peripheral inflammatory cells into the central nervous system (CNS) following JE virus (JEV). The JEV, a zoonotic pathogen transmitted by mosquitoes, is a single-stranded, positive-sense RNA virus. It is endemic to the Asia–Pacific region, encompassing countries such as China, India, and northern Australia [1]. Recently, the presence of JEV competent vectors have been documented in European regions such as Germany [2]. Furthermore, the inter-porcine transmission of JEV in mosquito-free environments has amplified the risk associated with viral spread and the potential for sustained infections [3]. Consequently, these facts underscore the emerging public health concerns associated with JEV. In the context of human infection, JEV exhibits a clinical spectrum that spans from mild febrile illness to severe meningoencephalitis, with an alarming annual tally of nearly 70,000 documented fatal cases [4]. Although the majority of JEV infection in endemic regions resent as mild febrile or subclinical illnesses, which subsequently confer protective immunity, it is noteworthy that approximately 25–30% of JE cases, primarily affecting infants, result in fatality, and 50% of cases lead to enduring neuropsychiatric complications [1]. Thus, JEV carriers a higher fatality rate compared to West Nile virus (WNV) infection, which is associated with a fatality rate of 3–5% (1100 deaths out of 29,000 symptomatic infections) [5]. In response to this critical health challenge, vaccination programs have been instituted in regions where JEV is endemic, aimed at mitigating the risk [6].

Significant advancements have been achieved in comprehending the kinetics and mechanisms governing JEV disseminations in hosts and JE pathogenesis using murine models [7–9]. Upon peripheral inoculation of the virus via mosquito bites, JEV undergoes initial replication with peripheral monocytes/macrophages and dendritic cells (DC), subsequently infiltrating the CNS by breaching the BBB [8, 10]. JE is considered as a neurologically and immunopathologically driven neuroinflammation, primarily instigated by an uncontrolled hyperimmune responses ensuing from viral incursion into the CNS [11, 12]. While JEV-specific T cells and virus-neutralizing IgM and IgG collectively eliminate the virus from both peripheral lymphoid tissues and the CNS, the innate immune responses appear to assume a pivotal role in the early containment of JEV infection, largely due to the delayed onset of adaptive immunity [13–15]. Therefore, the role of innate immune cells participating in the innate immune responses, including the type I IFN (IFN-I) innate response, is deemed indispensable for the regulation of JE progression following JEV infection.

Macrophages represent a prominent subset of target cells during the initial infection phase of JEV, and are acknowledged as a heterogenous population involved in multiple disease processes [16, 17]. Prior investigation has documented the ability of JEV to infect monocytes as undifferentiated macrophages at site of infection, thereby leading to viral dissemination to diverse tissues including the brain [18]. Consequently, it is assumed that macrophages play a critical role in instigating neuroinflammation in the CNS following JEV infection. Notably, Ly-6C ^+^ monocytes in blood exhibit migration into the brain, where they subsequently differentiate into inflammatory DCs, macrophages, and microglia [19, 20]. The infiltration of Ly-6C^ +^ monocytes in the CNS may potentially contribute to the pathophysiological aspects of severe neuroinflammation [17]. The plasticity stands out as a prominent characteristic of macrophages, exhibiting the capacity to maintain diverse phenotypes, thereby giving rise to various subpopulation. Therefore, it has been postulated that macrophages exhibit remarkable plasticity, enabling them to adeptly respond to environmental cues by altering their phenotype and physiological characteristics in response to cytokines and microbial signals [21, 22]. These alterations can give rise to distinct cell populations characterized by the production of either proinflammatory or anti-inflammatory cytokines. On the basis of the T helper type 1 (Th1) and Th2 polarization [23], macrophage populations are typically classified as M1 (classical) macrophages, which are responsible for producing proinflammatory cytokines, mediating pathogen resistance, and contributing to tissue damage, and M2 (alternative) macrophages, which produce anti-inflammatory cytokines, promote tissue repair and remodeling, and even play a role in tumor progression [24–26]. The polarization of macrophages and the subsequent immunopathological outcomes has yielded contradictory findings in the context of JE progression following JEV infection. Indeed, JEV was postulated to induce classical M1 activation of microglia that drives the production of proinflammatory cytokines, thereby contributing to severity of viral encephalitis [27, 28]. Conversely, JEV has been observed to modulates suppressors of cytokine signaling (SOCS)1 and 3 expression in macrophages to bring about changes in the JAK-STAT signaling cascade, so as to inhibit proinflammatory cytokine release [29]. Therefore, the clarification of mechanisms governing macrophage polarization and the comprehensive understanding of the immunopathological contribution to JE progression following JEV infection at both molecular and cellular levels could yield significant benefits in terms of JE control and therapeutic intervention.

Reactive oxygen species (ROS) are highly reactive molecules generated within different subcellular compartments, including endosomes via the NOX2-containing nicotinamide adenine dinucleotide phosphate (NADPH) oxidase (NOX) during an immune response, as well as in mitochondrial during cellular respiration [30]. Different cell types, such as epithelial cells, endothelial cells, and inflammatory cells like macrophages, monocytes, and neutrophils, express various isoforms of NOX [31]. NOX2 isoform is predominantly found in the macrophages and neutrophils, while both NOX1 and NOX2 isoforms are present in epithelial cells [32]. One established role of ROS generated by NOX2 is to defend the cells against invading bacterial and viral pathogens. Thus, NOX2-mediated ROS production within the endosomes of macrophages has been proposed to promote innate inflammation in response to various viral infection, such as influenza A virus and SARS-CoV [33–36]. In contrast, some viruses, including herpesviruses, thrives despite the induction of ROS, suggesting that ROS are beneficial for the virus [37]. Furthermore, ROS generated by NOX have the capacity to modulate macrophage polarization, specifically favoring M2 macrophage polarization [38]. This suggests that ROS produced by endosome NOX may influence innate immune responses by regulating macrophage polarization.

In this study, we investigated the role of ROS generated by NOX in JE progression. Intriguingly, our findings demonstrated a significant increase in resistance to JE progression following JEV infection in NOX2-deficient mice, which are unable to produce ROS. Additionally, NOX2-deficient mice exhibited a marked accumulation of M1-polarized macrophages in extraneural inflammatory and lymphoid tissues, accompanied by a reduction in viral load. Macrophages derived from NOX2-deficient mice displayed M1-polarized phenotypes after JEV infection, effectively suppressing JEV replication. Finally, the oral administration of the ROS scavenger enhanced resistance to JE progression. These results paradoxically suggest that ROS production by NOX2 exacerbates JE progression by facilitating JEV replication in extraneural tissues through the inhibition of M1 macrophage polarization.

## Materials and methods

### Ethics statement

All animal experiments described in the present study were conducted at Jeonbuk National University according to the guidelines set by the Institutional Animal Care and Use Committee (IACUC) of Jeonbuk National University, and were pre-approved by the Ethics Committee for Animal Experiments of Jeonbuk National University (Approval number: CBNU-2019–00079). The animal research protocol used in this study followed the guidelines set up by the nationally recognized Korea Association for Laboratory Animal Sciences (KALAS). All experimental protocols requiring biosafety were approved by the Institutional Biosafety Committee (IBC) of Jeonbuk National University and were performed in a biosafety cabinet at the Core Facility Center for Zoonosis Research, Jeonbuk National University.

### Animals, cells, and viruses

Wild-type (WT) C57BL/6 (H-2^b^) control mice (5–6 weeks old) were purchased from SAMTAKO (Osan, Korea) or Damool Science (Daejeon, Korea), and NOX2-deficient (NOX2 KO, H-2^b^; B6.129S-*Cybb*^*tm1Din*^/J, Stock No. 002365) mice were obtained from The Jackson Laboratory (Bar Harbor, ME, USA) [39]. OT-II transgenic (Tg) mice, which express αβ TCR specific for chicken ovalbumin 323–339 peptide (OVA_323-339_) of CD4 ^+^ T cells in the context of I-A^b^, were also obtained from The Jackson Laboratory. The JEV Beijing-1 strain was propagated in a mosquito cell line C6/36 (American Type Culture Collection (ATCC), CRL-1660) and passaged BHK-21 (ATCC, CCL-10) four times using DMEM supplemented with 2% FBS, penicillin (100 U/ml), and streptomycin (100 U/ml) as described previously [18, 40]. Virus stocks were titrated using focus-forming assay in Vero cells (ATCC, CCL-81) and stored in aliquots at − 80 °C until use.

### Mouse model of JE

WT and NOX2 KO mice were infected with JEV (7.5 × 10^7^ focus-forming units (ffu) via the intraperitoneal route (200 μl). Infected mice were monitored daily for mortality, morbidity (weight loss), and neurological disorders (limb paralysis, not moving but responsive). Infected mice were also scored daily for encephalitis signs and symptoms as described previously [9]. The encephalitis score represented a progressive range of behaviors from 1 to 5: (1) hunched, ruffled fur; (2) altered gait, slow movement; (3) immobile but responsive; (4) moribund and no response; and (5) death. In addition, clinical signs of disease were divided into seven grades which included subclinical, crumpled fur, hindlimb weakness, mild paralysis (rear leg weakness or paresis), moderate paralysis (both front legs and hind legs paresis), severe paralysis and moribund [41].

### Antibodies and reagents

The following mAbs were obtained from eBioscience (San Diego, CA, USA) or BioLegend (San Diego, CA, USA) for FACS analysis and other experiments: FITC-labeled anti-CD4 (RMA4-5), CD45 (30-F11), CD80 (16-10-A1), CD86 (GL1), MHC II (M5/114.15.2), CD44 (IM7), CD69 (H1.2F3), CD62L (MEL-14) and CD11b (M1/70); PE-labeled anti-CD8 (53–6.7), F4/80 (BM8), and IFN-γ (XMG1.2), TLR2 (6C2), CD154 (MR1), CD25 (PC61.5); PerCP/Cy5.5-labeled anti-mouse CD11b (M1/70), Ly-6C antibody (HK1.4), and IFN-γ (XMG1.2); PE/Cy7-labeled anti-IL-2 (JES6-5H4); APC-labeled anti-Ly6-G (1A8), IL-12/23p40 (C17.8), iNOS (CXNFT), TNF-α (MP6-XT22), CD206 (MR6F3), and CXCR3 (CXCR3-173). PE-labeled anti-mouse Tmem119 (106–6) was obtained from Abcam (Cambridge, MA, USA). The JEV epitope peptide of CD4^+^ T cells (NS1_132-145_ [TFVVDGPETKECPD]; NS3_563-574_ [WCFDGPRTNAIL]) or CD8^+^ T cells (NS4B_215-223_ [SAVWNSTTA]) was chemically synthesized at Peptron (Daejeon, Korea). Phorbol-12-Myristate-13-Acetate (PMA), ionomycin, and lipopolysaccharide (LPS) were purchased from Sigma-Aldrich (St. Louis, MO, USA).

### Quantitative real-time RT-PCR for determination of viral burden and cytokine expression

Viral burden and cytokine/chemokine expression in inflammatory and lymphoid tissues were determined via SYBR Green-based real-time qRT-PCR. Mice were i.p. infected with JEV (7.5 × 10^7^ ffu) and various tissues including spleen, spinal cord, and brain were harvested at different time points post-infection (pi). Total RNAs were extracted from the collected tissues using easy-BLUE (iNtRON, Inc., Daejeon, Korea) and subjected to real-time qRT-PCR using a CFX96 Real-Time PCR Detection system (Bio-Rad Laboratories, Hercules, CA, USA). Following reverse transcription of total RNA with High-Capacity cDNA Reverse Transcription Kits (Applied Biosystems, Foster, CA, USA), the reaction mixture (20 μl total) contained 2 μl of template cDNA, 10 μl of 2 × SYBR Premix Ex Taq, and 200 nM primers (Table 1). These reactions were denatured at 95 °C for 30 s and then subjected to 45 cycles of 95 °C for 5 s and 60 °C for 20 s. After completion of the reaction cycle, the temperature was increased from 65 °C to 95 °C at the rate of 0.2 °C/15 s, and fluorescence was measured every 5 s to construct a melting curve. A control sample lacking template DNA was run with each assay. All measurements were performed at least in duplicate to ensure reproducibility. The authenticity of the amplified product was determined by melting curve analysis. All data were analyzed using Bio-Rad CFX Manager, version 2.1 analysis software (Bio-Rad Laboratories). The expression of cytokines and chemokines was normalized to the levels of housekeeping gene β-actin. Viral burden was expressed by the copy number of viral RNA per microgram of total RNA after calculating the absolute copy number of viral RNA in comparison with the standard cDNA template of viral RNA.

**Table 1:** Specific primers for cytokines, chemokines, and transcription factors used in quantitative RT-PCR

Gene name^a^	Primer sequence (5′–3′)^b^	Position cDNA	Gene bank ID
*IL-6*	FP: AAC GAT GAT GCA CTT GCA GA RP: GAG CAT TGG AAA TTG GGG TA	313–332 576–595	NM_031168.2
*TNF-α*	FP: CGT CGT AGC AAA CCA CCA AG RP: TTG AAG AGA ACC TGG GAG TA	449–468 579–598	NM_013693.3
*IL-1β*	FP: AAG TGA TAT TCT CCA TGA GCT TTG T RP: TTC TTC TTT GGG TAT TGC TTG G	554–578 698–719	NM_008361.4
*CCL2*	FP: AAA AAC CTG GAT CGG AAC CAA RP: CGG GTC AAC TTC ACA TTC AAA G	347–367 426–447	NM_011333
*CCL5*	FP: CCT GCT GCT TTG CCT ACC TCT RP: ACA CAC TTG GCG GTT CCT TCG A	152–173 276–255	NM_009917
*CXCL2*	FP: ATC CAG AGC TTG AGT GTG ACG C RP: AAG GCA AAC TTT TTG ACC GCC	194–215 263–283	NM_009140.2
*IL-12p40*	FP: GGA AGC ACG GCA GCA GAA TA RP: AAC TTG AGG GAG AAG TAG GAA TGG	686–705 842–865	NM_001303244.1
*iNOS*	FP: AAC GGA GAA CGT TGG ATT T RP: CAG CAC AAG GGG TTT TCT TC	212–228 342–358	NM_010927.4
*CXCL9*	FP: TGC ACG ATG CTC CTG CA RP: AGG TCT TTG AGG GAT TTG TAG TGG	137–153 176–199	NM_008599.4
*Arg1*	FP: GGC AAC CTG TGT CCT TTC TC RP: ACA CGA TGT CTT TGG CAG AT	527–546 609–628	NM_007482.3
*IL-4Ra*	FP: ATC TGC GTG CTT GCT GGT TCT RP: CTG GTA TCT GTC TGA TTG GAC CG	118–138 533–555	NM_001008700.4
*CD206*	FP: TCT TTG CCT TTC CCA GTC TCC RP: TGA CAC CCA GCG GAA TTT C	45–65 267–285	NM_008625.2
*Fizz1*	FP: TCC CAG TGA ATA CTG ATG AGA RP: CCA CTC TGG ATC TCC CAA GA	101–121 295–314	NM_020509.4
*Ym1*	FP: GCA GAA GCT CTC CAG AAG CAA TCC TG RP: ATT GGC CTG TCC TTA GCC CAA CTG	10–35 130–153	NM_009892.3
*IRF4*	FP: GA GCT GCA AGT GTT TGC TCA CCA T RP: AC AGT TGT CTG GCT AGC AGA GGT	1194–1217 1313–1335	NM_013674.2
*IRF5*	FP: GGA AGA AAT GAA GCC AGC AG RP: ACC CTG GGG TAA TTG GAC TC	1931–1950 2001–2020	NM_001252382.1
*β-actin*	FP: TGG AAT CCT GTG GGA TCC ATG AAA C RP: TAA AAC GCA GCT CAG TAA CAG TCC G	915–939 1239–1263	NM_007393.5
*JEV*	FP: GGC TTA GCG CTC ACA TTC A RP: GCT GGC CAC CCT CTC TTC TT	4132–4150 4207–4226	AB920399.1

^a^IL, interleukin

^b^FP, forward primer; RP, reverse primer

### Histopathological examinations

Histopathological examination was performed using brains derived from WT and NOX2 KO mice infected with JEV. Brains were embedded in paraffin at 5 dpi, and 10-μm sections were prepared and stained with H&E. The slides were scanned and analyzed with a slide scanner (Motic Digital Pathology, Kowloon, Hong Kong). Inflammation was scored according to the extent of infiltration by inflammatory cells, as previously delineated on a scale from 0 to 3: 0, no inflammatory cells; (1) few scattered inflammatory cells; (2) organization of inflammatory infiltrates around blood vessels; and (3) extensive perivascular cuffing with extension into adjacent parenchyma [42].

### Cytokine ELISA

The levels of IL-6, TNF-α, and IL-12p40 proteins in sera or culture media were determined by sandwich ELISA, according to the manufacturer’s protocols (ThermoFisher, Waltham, MA, USA). Nunc MaxiSorp flat-bottom 96-well plates (ThermoFisher) were coated with 50 µl of specified working concentration of capture antibodies in 1 × coating buffer and incubated overnight at 4 °C. Plates were washed three times with ELISA washing buffer (PBS containing 0.05% Tween 20; PBST), and then blocked with 50 µl ELISA diluent (ELISA/ELISpot diluent, Invitrogen) for at least 2 h at 37 °C. The loading of sera or culture media to the plates was proceeded with standards for recombinant cytokine proteins, after which the plates were kept overnight at 4 °C. Following another washing, the specified concentration of biotin-conjugated detection antibodies was added, and incubated at 37 °C for 1 h. The plates were washed with PBST, followed by incubation with streptavidin-horse radish peroxidase (HRP) at 37 °C for 1 h. Color development was carried out by addition of tetramethylbenzidine (TMB) substrate solution of 50 µl and the reaction was stopped by addition of 25 µl stop solution (1 M phosphoric acid). OD values were measured at 450 nm wavelength by ELISA reader (Molecular Device, San Jose, CA, USA). Cytokine concentrations were calculated with SoftMax Pro 3.4, depending on comparison with two concentrations of standard cytokine proteins.

### Analysis of infiltrated leukocytes in the CNS

Mice infected with JEV were perfused with 30 ml of HBSS at 3 and 5 dpi via cardiac puncture of the left ventricle. Brains were then harvested and homogenized by gently pressing them through a 100-mesh tissue sieve, followed by digestion with 5 mg/ml of collagenase type IV (Worthington Biochem, Freehold, NJ, USA), 10 mg/ml DNase I (Amresco, Solon, OH, USA), and incubation with RPMI medium for 1 h at 37 °C with shaking. Cells were separated by centrifugation at 800 × *g* for 30 min (Axis-Shield, Oslo, Norway) using Opti-prep density gradient (18/10/5%), and the cells were collected from 18 to 10% interface and washed twice with PBS. The cells were then counted and stained for CD11b, Ly-6C, and Ly-6G with directly conjugated antibodies for 30 min at 4 °C. Finally, cells were fixed with 1% formaldehyde. Data collection and analysis were performed using a FACS Calibur flow cytometer (Becton Dickson Medical Systems, Sharon, MA, USA) with FlowJo software (Tree Star, San Carlos, CA, USA).

### JEV-specific humoral and T cell responses

JEV-specific CD4^+^ and CD8^+^ T cell responses were determined by intracellular IFN-γ and TNF-α staining in response to stimulation with JEV epitope peptides, as described previously [9, 43]. Surviving mice infected with JEV (7.5 × 10^7^ ffu/mouse) were euthanized on day 7dpi and leukocytes were prepared from the spleen. The splenocytes were then cultured in 96-well culture plates (5 × 10^5^ cells/well) in the presence of synthetic peptide epitopes (NS1_132–145_, NS3_563-574_, and NS4B_215–225_) for 12 h and 6 h to observe CD4 ^+^ and CD8 ^+^ T cell responses, respectively. Monensin at concentration of 2 μM was added to antigen-stimulated cells 6 h before harvest. Cells were washed twice with FACS buffer containing monensin, surface-stained with FITC-anti-CD4 or CD8 antibodies for 30 min at 4 °C, and then washed twice with PBS containing monensin. After fixation, cells were washed twice with 1 × Permeabilization Buffer (eBioscience) and stained with PepCP-Cy5.5 anti-IFN-γ or APC-anti-TNF-α in the permeabilization buffer for 30 min at room temperature. Finally, cells were washed twice with PBS and fixed using the fixation buffer. CD4^+^Foxp3^+^ Treg cells were enumerated by intracellular Foxp3 staining, combined with surface CD4 molecule. Sample analysis was performed using a FACS Calibur flow cytometer (Becton Dickson Medical Systems) with FlowJo software (Tree Star).

### Analysis of M1 macrophage polarization

M1 macrophage polarization was ex vivo evaluated by determining IL-12p40 and iNOS production in response to brief stimulation with LPS using leukocytes prepared from peritoneal cavity and spleen [44, 45]. WT and NOX2 KO mice infected with JEV were euthanized to harvest the leukocytes from peritoneal cavity and spleen. Cells of peritoneal cavity were harvested using solution with mixed Hank’s balanced salt solution (HBSS) with 2% FBS, and washed with PBS twice. Splenocytes were harvested with RPMI medium containing 10% FBS and homogenized by pressing them on 100-µm tissues sieve, and centrifugated at 800 ×*g* for 6 min. Following brief stimulation with LPS, the cells were counted and stained for surface CD45, CD11b, and F4/80 followed by intracellular IL-12p40 and iNOS staining. For M1 polarization of macrophages derived from bone marrow cells (BMDM), bone marrow (BM) cells were prepared from femur and tibia of the adult mice (WT and NOX2 KO mice, 6–8 weeks old), filtered to remove bone fragments with cell strainer. BM cells were then washed, resuspended, and incubated in culture medium with GM-CSF (10 ng/ml). GM-CSF was added 4 days later once more. Following seven days, adherent BMDM were harvested and seeded at 1 × 10^6^ cell/well in 6-well plates. After overnight incubation, the medium was replaced with fresh medium containing LPS (100 ng/ml) and murine IFN-γ (10 ng/ml) (Peprotech, Cranbury, NJ, USA) for generation of M1 macrophages. The cells were incubated under this condition for different time periods, depending on experiments. The monolayer of BMDM which was unpolarized or M1 polarized were harvested and used for further analyses including IL-12p40 and iNOS production and real-time qRT-PCR for M1 effector molecule expression.

### Ag-presentation of BMDM derived from the BM cells

Ag-presentation of unpolarized and M1-polarized BMDM derived from WT and NOX2 KO mice was evaluated by the presentation capability of OVA_323-339_ peptide-loaded BMDM to OT-II CD4 ^+^ T cells. Briefly, CD4 ^+^ T cells were purified from OT-II Tg mice using MojoSort isolation (Biolegned, San Diego, CA, USA), according to the manufacturer’s instruction, after which they were co-cultured with different ratio of unpolarized or M1-polarized BMDM in the presence of OVA_323–339_ peptide (1 µg/ml). The culture was then incubated for 24 h at 37 °C in a humidified 5% CO_2_ incubator. Following harvesting cells in co-culture system, the cells were intracellularly stained with anti-mouse IL-2 antibody, combined with surface CD4 staining. The activation levels of OT-II CD4 ^+^ T cells stimulated with OVA_323–339_ peptide-loaded BMDM were also evaluated by staining surface markers (CD44, CD154, CD25, CD69, CD62L). Data collection and analysis were performed using a FACS Calibur flow cytometer (Becton Dickson Medical Systems) with FlowJo software (Tree Star).

### Determination of ROS levels in sera and macrophages

ROS levels in sera were assessed using ROS ELISA method. Briefly, the serum samples of JEV-infected mice were collected 2 dpi, and ROS levels in sera were subsequently quantified by ROS ELISA kit (Abbkine, USA) in accordance with the protocol provided. Intracellular ROS levels in macrophages were detected using total ROS assay kit (Invitrogen, Carlsbad, CA) after brief stimulation of the leukocytes derived from peritoneal cavity and spleen with LPS. Leukocytes prepared from peritoneal cavity and spleen were stained with ROS-assay stain solution of kit and incubated for 1 h at 37 °C. ROS assay stain-labeled cells were subsequently stimulated with LPS (200 ng/ml) for 6 h, after which they were subjected to flow cytometric analysis. The fluorescence intensity of ROS assay stain in CD11b ^+^ F4/80 ^+^ macrophages was assessed using a FACS Calibur flow cytometer (Becton Dickson Medical Systems) with FlowJo software (Tree Star).

### Statistical analysis

All data were expressed as average ± standard error of the mean (SEM). Statistically significant differences between groups were analyzed using an unpaired two-tailed Student’s *t*-test for ex vivo experiments and immune cell analysis. For multiple comparisons, statistical significance was determined using one-way or two-way analysis of variance (ANOVA) with repeated measures followed by Bonferroni post hoc tests. Statistical significance of viral burden and in vivo cytokine gene expression were evaluated by Mann–Whitney test or unpaired two-tailed Student’s *t*-test. Kaplan–Meier survival curves were analyzed by log-rank test. A *p*-value ≤ 0.05 was considered significant. All data were analyzed using GraphPadPrism4 software (GraphPad Software, Inc., San Diego, CA, USA).

## Results

### NOX2 ablation attenuates JE progression

**Fig. 1:**
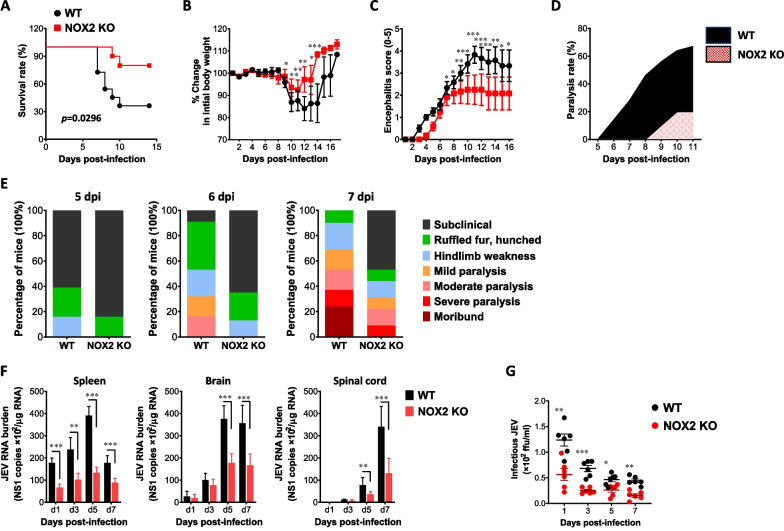
NOX2-ablated mice show enhanced resistance to JE. **A**–**C** Susceptibility to NOX2-ablated mice to JE. Wild-type (WT) and NOX2 KO mice (5 to 6 weeks old, n = 10–11) were inoculated i.p. with JEV (7.5 × 10^7^ ffu) and examined over 14 days after their survival, body weight, and encephalitis. **A** Curve showing survival rate; **B** Changes in body eight; **C** Encephalitis score. **D** Ratio of mice showing neurological disorders during JE progression. Mice infected with JEV were examined every 6 h from 5 to 11 dpi. **E** Clinical signs. Clinical signs of JEV-infected WT and NOX2 KO mice were monitored and scored on day 5, 6, and 7 post-infection. **F** Viral burden in lymphoid and inflammatory tissues during JE progression. Viral burden in lymphoid (spleen) and inflammatory tissues (brain and spinal cord) of infected mice (n = 5–6) were assessed by real-time qRT-PCR at the indicated time points. Viral RNA load was expressed as viral RNA copy number targeted on JEV NS1 gene per microgram of total RNA. **G** Infectious JEV burden in sera. Following JEV infection, the levels of infectious JEV were determined by a focus-forming assay using sera collected at the indicated time points. Data show the mean ± SEM of the levels derived from at least 2 individual experiments (n = 5–6). *p < 0.05, **p < 0.01, ***p < 0.001 between the levels derived from WT and NOX2 KO mice

The role of NOX2, a major enzyme responsible for the production of ROS, has not been elucidated in viral encephalitis following neurotrophic viruses such as JEV and WNV. While various reports have indicated contrasting alterations in resistance to viral infections due to NOX2 deficiency or inhibition [46], there have been no attempts to investigate NOX2 role in the CNS inflammation disorder induced by actual JEV infection. In order to address this issue of NOX2 in JE, we first examined the susceptibility of NOX2-deficient (NOX2 KO) mice to viral encephalitis caused by JEV infection. After WT and NOX2 KO mice were infected with JEV, surviving mice were monitored until 14 dpi (Fig. 1A). Mice in both groups showed comparable clinical signs, starting with generalized piloerection, paresis, and rigidity, followed by progression into severe neurological signs such as postural imbalance, ataxia, and generalized tonic–clonic seizure from 6 to 10 dpi. However, somewhat interestingly, NOX2 KO mice showed enhanced resistance to JE with decreased mortality after showing neurological disorders (70% mortality for WT mice vs. 15% mortality for NOX2 KO mice). Likewise, NOX2 KO mice showed reduction in loss of body weight in the course of JE (Fig. 1B), and delayed and reduced encephalitis progression was observed in NOX2 KO mice, compared to WT mice (Fig. 1C). In support of this finding, NOX2-ablated mice showed delayed and reduced proportion of neurological disorders starting from 8 dpi, compared to WT mice showing neurological disorder started from 5 dpi (Fig. 1D). Thus, this result clearly indicates that NOX2 ablation results in enhanced resistance to JE with reduction of neurological disorder presentation. To clarify clinical signs of reduced JE in NOX2 KO mice, we monitored JEV-infected mice for clinical signs depending on seven signs in the course of JE. NOX2 KO mice developed obviously less severe clinical signs from 5 to 7 dpi, compared to WT mice (Fig. 1E). To further examine the enhanced resistance of NOX2 KO mice to JE progression, we assessed viral burden within lymphoid and the CNS tissues (Fig. 1F). NOX2 KO mice were found to exhibit five fold decreased viral load in the spleen, brain, and spinal cord, which indicates that NOX2 ablation induces the reduction of JEV replication in lymphoid and the CNS tissues. Similarly, we found that NOX2 KO mice exhibited lower levels of infectious JEV in sera compared to WT mice (Fig. 1G). Therefore, these findings paradoxically suggest that ROS produced by NOX2 could exacerbate JE progression with contributing to enhanced JEV replication.

### NOX2 ablation attenuates neuroinflammation caused by JEV infection

**Fig. 2:**
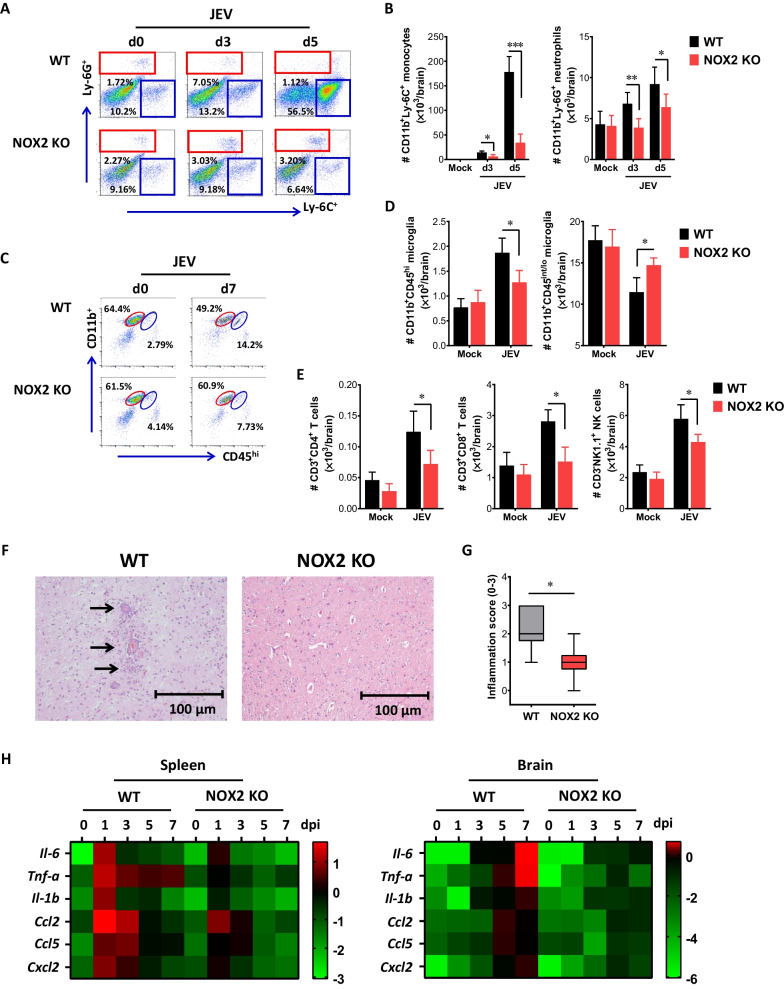
Ameliorated JE progression in NOX2-ablated mice. **A**, **B** The frequency and number of Ly-6C ^+^ monocytes and Ly-6G ^+^ neutrophils in the brain. Wild-type (WT) and NOX2 KO mice were inoculated with JEV (7.5 × 10^7^ ffu), and the frequency (**A**) and total number (**B**) of Ly-6C ^+^ monocytes and Ly-6G ^+^ neutrophils in the brain were determined by flow cytometric analysis at 3 and 5 dpi using vigorous heart perfusion. Values in the dot-plots show the average percentage of each population after gating on CD11b ^+^ cells. **C** and **D** The frequency and number of activated and resting microglia in the brain. The frequency (**C**) and total number (**D**) of CD11b^+^CD45^hi^ (activated microglia) and CD11b^+^CD45^int/lo^ (resting microglia) cells were determined by flow cytometric analysis at 7th dpi. (**E**) Total number of lymphoid cells in the brain. The total number of lymphoid cells (CD4 ^+^ and CD8 ^+^ T cells, NK cells) were assessed on the 7th dpi. **F** Histopathological examinations of the brain tissues derived from infected WT and NOX2 KO mice. Brain sections of WT and NOX2 KO mice were prepared and stained with H&E 5 days after JEV infection via intraperitoneal route. Representative photomicrographs of the brain were obtained from blood vessel areas, meninges, and ventricles. **G** Inflammatory score of the brain tissues derived from WT and NOX2 KO mice. Inflammation was scored based on the degree of infiltration of inflammatory cells at 5th dpi. **H** The attenuated expression of inflammatory cytokines and chemokines in NOX2 KO mice. The expression of cytokines and chemokines in the spleen and brain was determined by real-time qRT-PCR at the indicated time points after WT and NOX2 KO mice were inoculated i.p. with JEV (7.5 × 10^7^ ffu). The expression of cytokines and chemokines was normalized to β-actin expression and displayed as the average of at least four independent samples, according to the indicated color on a log_2_ scale. Data show the mean ± SEM of the levels derived from at least 2 individual experiments (n = 5–6). *p < 0.05, **p < 0.01, ***p < 0.001 between the levels derived from WT and NOX2 KO mice

CNS-infiltration of Ly-6C ^+^ monocytes and Ly-6G ^+^ neutrophils derived from the myeloid cells represents a hallmark neuroinflammation in the progression of JE [47]. To further characterize the CNS inflammation in NOX2 KO mice following JEV infection, we assessed the infiltration of CD11b ^+^ Ly-6C ^+^ monocytes and CD11b ^+^ Ly-6G ^+^ neutrophils. Our results revealed a significantly lower frequency of infiltrated Ly-6C ^+^ monocytes and Ly-6G ^+^ neutrophils in the brain of NOX2 KO mice at 3 and 5 dpi, compared to that observed in the brain of WT mice (Fig. 2A). Notably, NOX2 KO mice displayed greatly reduced infiltration of Ly-6C ^+^ monocytes at 5 dpi, compared to WT mice. Similarly, the absolute number of infiltrated Ly-6C ^+^ monocytes and Ly-6G ^+^ neutrophils in the brain of NOX2 KO mice decreased two- and five-fold at 5 dpi, respectively (Fig. 2B). In particular, the reduction in CNS-infiltration of Ly-6C ^+^ monocytes was more pronounced in NOX2 KO mice compared to Ly-6G ^+^ neutrophils. The involvement of microglia cells in the pathogenesis of encephalitis induced by certain neurotrophic viruses, such as WNV, has been demonstrated [48, 49]. Consequently, triple-color staining (CD11c/CD11b/CD45) was employed to discern between the resting and activated microglia. Based on the CNS myeloid cell classification of Ford et al. [50], we observed diminished frequency and absolute count of activated CD11b ^+^ CD45^hi^ microglia in the brain of NOX2 KO mice following JEV infection. Conversely, resting CD11b ^+^ CD45^int/lo^ microglia cells exhibited a modest decrease in NOX2 KO mice compared to WT mice (Fig. 2C and D). Additionally, NOX2 KO mice displayed a sustained reduction in the number of CD4 ^+^ , CD8 ^+^ T cells, and NK cells in the brain following JEV infection, in contrast to WT mice (Fig. 2E). These results indicate that NOX2 ablation leads to a reduction in the infiltration of inflammatory cells from the peripheral sites following JEV infection. To better understand less CNS inflammation in NOX2 KO mice, histopathological examinations were performed. Histopathological examinations revealed that decreased BBB permeability in JEV-infected NOX2 KO mice was associated with reduced observation of perivascular cuffing, compared to those of WT mice (Fig. 2F). Consistent with this observation, NOX2 KO mice exhibited a decreased inflammation score in the brain, as determined by the extent of inflammatory cell infiltration (Fig. 2G). Hence, these results suggest that the absence of NOX2 leads to a milder progression of CNS inflammation, marked by a reduced influx of inflammatory cells from peripheral sites following JEV infection.

Viral encephalitis caused by neurotrophic viruses is indirectly derived from CNS degeneration, stemming from robust immunological responses characterized by uncontrolled secretin of cytokines and chemokines, thereby leading to the activation of microglia and astrocytes [11, 12]. In contrast, the proper expression of cytokines and chemokines in peripheral tissues is crucial for viral clearance, thereby reducing viral burden to invade the CNS tissues [8, 10, 51]. Thus, measuring the expression of cytokines and chemokines in peripheral lymphoid and CNS tissues can offer valuable insights into the progression of JE in NOX2 KO mice. To gain a deeper understanding of JE progression in NOX2 KO mice, we examined the expression of cytokines and chemokines in the primary target lymphoid organ, the spleen, and the brain, a key inflammatory CNS tissues, following JEV infection. We found that JEV infection in NOX2 KO mice led to significantly reduced expression of cytokines and chemokines in the peripheral lymphoid tissues (spleen) during the early stage (1–3 dpi) after JEV infection (Fig. 2H). Consequently, NOX2 KO mice exhibited lower expression of cytokine and chemokines in CNS tissues than WT mice during the later stage when neurological symptoms manifested. This outcome was likely influenced by the viral burden in spleen and brain. Furthermore, the reduced expression of chemokines, such as CCL2, CCL5, and CXCL2, in the brain of NOX2 KO mice was thought to contribute to the reduction of inflammatory cell infiltration in the brain, ultimately leading to less neuroinflammation.

### NOX2-ablated mice show enhanced JEV-specific T cell responses with reduced CD4 ^+^ Foxp3 ^+^ Treg number

**Fig. 3:**
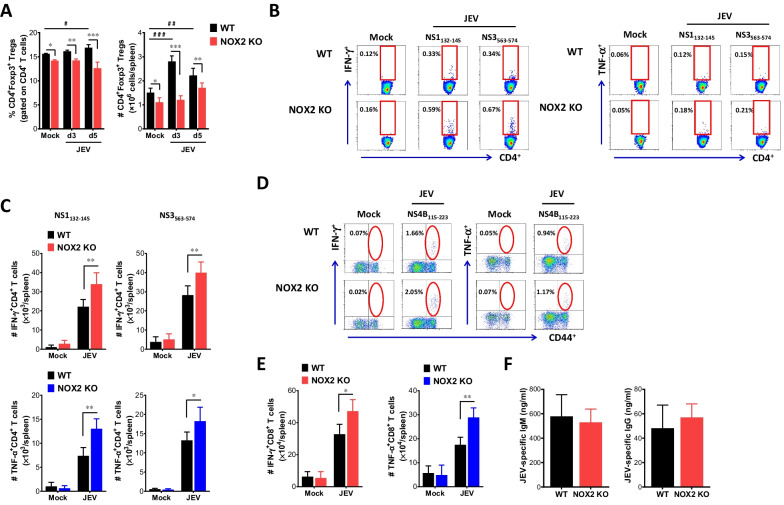
NOX2-ablated mice show enhanced JEV-specific T cell responses. **A** The frequency and total number of CD4 ^+^ Foxp3 ^+^ Tregs in the spleen of NOX2 KO mice. CD4 ^+^ Foxp3 ^+^ Tregs in the spleen of Wild-type (WT) and NOX2 KO mice were detected with intracellular Foxp3 and surface CD4 staining at 3 and 5 dpi. **B** and **C** JEV-specific CD4 ^+^ T cell responses. **D** and **E** JEV-specific CD8 ^+^ T cell responses. The splenocytes were prepared from surviving WT and NOX2 mice 7 days following infection with JEV and used for stimulation with JEV epitope peptide of CD4 ^+^ T cells (NS1_132-145_, NS3_563-574_) or CD8 ^+^ T cells (NS4B_215-223_) for 12 or 8 h, respectively. The frequency and absolute number of JEV-specific CD4 ^+^ and CD8 ^+^ T cells were determined by intracellular cytokine (IFN-γ and TNF-α) staining combined with surface CD4 and CD8 staining. **F** Serum levels of JEV E protein-specific IgM and IgG. Levels of JEV E protein-specific IgM and IgG in sera were determined by conventional ELISA using sera collected from surviving mice at 7 dpi. Values in representative dot-plots denote average percentage of indicated cell population. Bar charts show the mean ± SEM of the levels derived from at least 2 individual experiments (n = 5–6). *p < 0.05, **p < 0.01, ***p < 0.001 between the levels derived from WT and NOX2 KO mice. ^#^*p* < 0.05, ^##^*p* < 0.01, ^###^*p* < 0.001 between the levels derived from mock and JEV-infected mice

Antiviral adaptive immune responses, mediated by effector Ag-specific CD4 ^+^ and CD8 ^+^ T cells, are required for the regulation of JE progression by controlling and clearing JEV in extraneural tissues and the CNS [13, 14, 52]. When we examined the frequency and number of CD4 ^+^ Foxp3 ^+^ Treg cells in both WT and NOX2 KO mice, WT mice showed slightly increased frequency levels of CD4 ^+^ Foxp3 ^+^ Treg cells at 3 and 5 dpi, as previously described [52], but the frequency of CD4 ^+^ Foxp3 ^+^ Treg cells was not changed in NOX2 KO mice (Fig. 3A). Conversely, NOX2 KO mice exhibited a reduction in the frequency of CD4 ^+^ Foxp3 ^+^ Treg cells in response to JEV infection. In support, NOX2 KO mice contained significantly decreased number of CD4 ^+^ Foxp3 ^+^ Treg cells in the spleen with the levels peaked at 3 dpi, compared to WT mice. Although some of both WT and NOX2 KO mice infected with JEV exhibited neurological disorders at 5–6 dpi, a time before fully induced functional adaptive immune responses, we examined the generation of JEV-specific CD4 ^+^ and CD8 ^+^ T cell responses in surviving WT and NOX2 KO mice at 7 dpi. Our results revealed that NOX2 KO mice exhibited significantly increased frequencies of JEV-specific CD4 ^+^ T cells producing IFN-γ and TNF-α upon stimulation with the epitope peptides NS1_132-145_ and NS3_563-574_, compared to those in WT mice (Fig. 3B). Also, IFN-γ ^+^ CD4 ^+^ and TNF-α ^+^ CD4 ^+^ T cell number were higher in the spleen of NOX2 KO mice upon stimulation with JEV CD4 ^+^ T cell epitope peptides at 7 dpi, compare to WT mice (Fig. 3C). Similarly, NOX2 KO mice exhibited significantly increased JEV-specific CD8 ^+^ T cell responses, as shown by higher frequency and number of IFN-γ and TNF-α-producing CD8 ^+^ T cells upon stimulation with CD8 ^+^ T cell epitope peptide NS4B_115–223_ (Fig. 3D and E). Therefore, the reduced population of CD4  Foxp3 ^+^ Treg cells is presumed to contribute to the heightened Th1 CD4 ^+^ and CD8 ^+^ T cell responses producing IFN-γ and TNF-α upon JEV infection. These responses may play a role in controlling JE progression in NOX2 KO mice by reducing viral burden at the periphery during later stage. In contrast with enhanced JEV-specific Th1 CD4 ^+^ and CD8 ^+^ T cell responses in NOX2 KO mice, JEV E protein-specific IgM and IgG in sera were found to be detected at similar levels in both NOX2 KO and WT mice (Fig. 3F), which indicate that NOX2 ablation has no discernible impact on the induction of JEV-specific humoral immune responses.

### Attenuated JE progression in NOX2-ablated mice is linked to reduced ROS production with the accumulation of M1-like macrophages

**Fig. 4:**
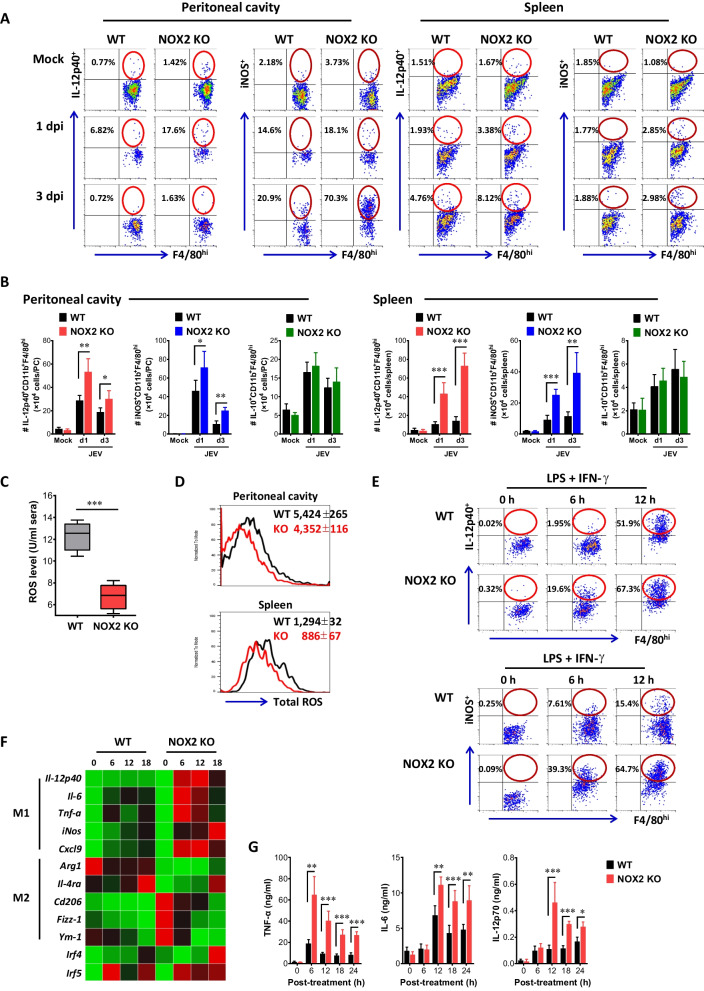
Attenuated JE progression is associated with increased accumulation of M1 macrophages in NOX2-ablated mice. **A** Increased accumulation of M1 macrophages in peritoneal cavity and spleen of NOX2 KO mice. **B** Accumulated number of M1 macrophages in peritoneal cavity and spleen. Leukocytes were prepared from the peritoneal cavity and spleen of Wild-type (WT) and NOX2 KO mice 1 and 3 dpi, and briefly stimulated with LPS (200 ng/ml) for 6 h. M1 macrophages were then detected by intracellular IL-12p40 and iNOS staining combined with surface CD11b and F4/80 staining. **C** Reduced ROS levels in sera of JEV-infected NOX2 KO mice. ROS levels were determined by ROS ELISA kit using sera derived from JEV-infected WT and NOX2 KO mice at 2nd dpi. **D** Intracellular ROS levels of CD11b ^+^ F4/80 ^+^ macrophages derived from WT and NOX2 KO mice. Intracellular ROS levels in macrophages derived from peritoneal cavity and spleen were assessed using total ROS assay kit 2 days after JEV infection. Values in histograms show the average MFI ± SEM of total ROS in macrophages population after gating on CD11b ^+^ F4/80 ^+^ cells. **E** Enhanced M1 polarization of NOX2-deficient macrophage. BMDM derived from BM cells of WT and NOX2 KO mice were stimulated with LPS and IFN-γ for 6 and 12 h to drive M1 polarization. The M1 polarization of BMDM was evaluated by intracellular IL-12p40 and iNOS staining combined with surface CD11b and F4/80 staining. **F** Increased expression of M1 effector molecules in NOX2-deficient macrophages. Total RNA extracted from M1-polarized BMDM driven from WT and NOX2 KO mice was used for determining the expression of M1 and M2 effector molecules with real-time qRT-PCR. The expression of M1 and M2 effector molecules was normalized to β-actin expression and displayed as the average of at least four independent samples, according to the indicated color on a log_2_ scale. **G** Higher production of M1 effector cytokines from NOX2-deficient macrophages. BMDM derived from WT and NOX2 KO mice was stimulated with LPS and IFN-γ form the indicated times and the production of M1 effector cytokines (TNF-α, IL-6, IL-12p70) was determined by cytokine ELISA using culture media. Data show the mean ± SEM of the levels derived from at least 2 individual experiments (n = 5–6). *p < 0.05, **p < 0.01, ***p < 0.001 between the levels derived from WT and NOX2 KO mice

Macrophages are heterogenous immune cells differentiated from Ly-6C ^+^ and Ly-6C- monocytes and play an important role in neuroinflammation caused by neurotrophic viruses [18–20]. After viral infection, Ly-6C + monocytes can differentiate into M1 or M2 macrophages at inflammation sites depending on surrounding microenvironment, and thereby impacting the progression of inflammation caused by viral infection [27, 28]. Recent findings have indicated that ROS can influence M1 or M2 macrophage polarization [38]. Therefore, we examined whether ROS produced by NOX2 ultimately contribute to macrophage polarization, and consequently played a role in the resistance of NOX2 KO mice to JE progression. To this end, we examined ex vivo polarization of macrophages derived from peritoneal cavity and spleen of JEV-infected WT and NOX2 KO mice in response to brief LPS stimulation. Our results revealed that NOX2 KO mice exhibited more enhanced accumulation of M1-polarized macrophages in the peritoneal cavity and spleen, compared to WT mice (Fig. 4A). Specifically, IL-12p40 and iNOS-positive M1-polarized CD11b ^+^ F4/80 ^+^ macrophages were detected in the peritoneal cavity and spleen of NOX2 KO mice with higher levels than WT in response to brief LPS stimulation. In support, NOX2 KO mice contained more accumulated number of IL-12p40 and iNOS-producing M1-polarized macrophages in the peritoneal cavity and spleen, compared to WT mice (Fig. 4B). However, the accumulated number of IL-10-producing M2 macrophages was comparable in both WT and NOX2 KO mice. Hence, this finding indicates that NOX2 deficiency leads to an increased accumulation of IL-12p40 and iNOS-producing M1-polarized macrophages in primary inflammation and lymphoid tissues during JE progression following i.p. infection with JEV. Moreover, our result revealed that the reduced ROS production in sera of NOX2 KO mice was closely linked to an increased accumulation of M1 macrophages (Fig. 4C). Also, CD11b ^+^ F4/80 ^+^ macrophages derived from the peritoneal cavity and spleen of NOX2 KO mice displayed lower production of total ROS compared to those derived from WT mice (Fig. 4D), suggesting a strong correlation between reduced ROS production in macrophages and enhanced M1 polarization. To further confirm the impact of NOX2 on macrophage polarization, we prepared BMDM derived from BM cells of WT and NOX2 KO mice using GM-CSF and induced to undergo M1 polarization through stimulation with LPS and IFN-γ [53, 54]. Consequently, BMDM derived from BM cells of NOX2 KO mice exhibited more enhanced M1 polarization, as shown by increased IL-12p40 and iNOS-producing CD11b ^+^ F4/80 ^+^ macrophages in response to stimulation with LPS and IFN-γ (Fig. 4E). In support, BMDM derived from NOX2 KO mice displayed higher expression of M1 effector molecules (IL-12p40, IL-6, TNF-α, iNOS, CXCL9), compared to those of WT mice (Fig. 4F). However, the expression of M2 effector molecules (IL-4Rα, CD206, Fizz-1, Ym-1) showed no changes in BMDM derived from NOX2 KO mice, which indicates that NOX2 ablation may not affect M2 macrophage polarization. Also, IRF5, which plays a significant transcription role in the de novo differentiation of M1 macrophage by GM-CSF [52], demonstrated sustained upregulation in BMDM derived from NOX2 KO mice. It was also curious that IRF4, involved in the de novo differentiation of M2 macrophages by M-CSF [52, 53], exhibited notably heightened expression in NOX2-deficient BMDM. In addition, BMDM derived from NOX2 KO mice was found to produce TNF-α, IL-6, and IL-12p70 with higher levels in culture media, compared to those of WT mice (Fig. 4G). Collectively, these results suggest that the reduced ROS production due to NOX2 ablation leads to enhanced M1 polarization of macrophages in the peripheral inflammatory sites (peritoneal cavity) and lymphoid tissues (spleen), thereby contributing to the control of JE progression in NOX2 KO mice.

### NOX2 ablation facilitates de novo differentiation of M1 macrophages to activate Th1 CD4 + T cells

**Fig. 5:**
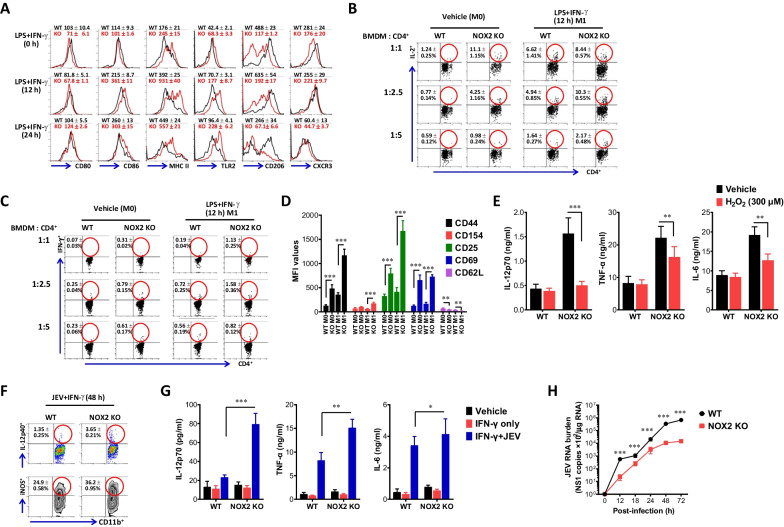
NOX2 is negative regulator of de novo differentiation of M1 macrophages. **A** The expression of M1 and M2 surface markers in NOX2-deficient BMDM. BMDM derived from BM cells of WT and NOX2 KO mice were stimulated with LPS and IFN-γ for 12 and 24 h to drive M1 polarization, and used for evaluating the expression of surface markers by flow cytometric analysis. Values in the histograms show the average MFI ± SEM of each surface molecule expression in macrophage population after gating on CD11b ^+^ F4/80 ^+^ cells. **B** and **C** Enhanced Ag-presentation of NOX2-deficient BMDM. BMDM derived from the BM cells of WT and NOX2 KO mice was either stimulated with (M1) or without (M0) LPS and IFN-γ for 12 h, and co-cultured with purified OT-II CD4 ^+^ T cells at the varying ratios in the presence of OVA_323-339_ peptide for 24 h and 72 h to assess the generation of IL-2 and IFN-γ-producing cells, respectively. The Ag-presentation of BMDM to OT-II CD4 ^+^ T cells was evaluated by intracellular IL-2 and IFN-γ staining combined with surface CD4 staining. Values in dot-plots show the mean ± SEM percentage of IL-2 and IFN-γ-producing CD4 ^+^ T cells after gating on CD4 ^+^ cells. **D** Enhanced activation of OT-II CD4 ^+^ T cells stimulated with NOX2-deficient BMDM. The activation markers of OT-II CD4 ^+^ T cells stimulated with BMDM derived from WT and NOX2 KO mice were determined by flow cytometric analysis after surface staining with CD4 and activation marker antibodies. Data was expressed by the average MFI ± SEM levels of each activation marker. **E** Suppression of M1 macrophage polarization by NOX2-generated H_2_O_2_. BMDM derived from WT and NOX2 KO mice was stimulated with LPS and IFN-γ in the absence or presence of H_2_O_2_ (300 µM) for 18 h. The production of M1 effector cytokines (IL-12p40, TNF-α, IL-6) was determined by cytokine ELISA using culture media. **F** Facilitated de novo differentiation of M1 macrophage by JEV infection. BMDM derived from BM cells of WT and NOX2 KO mice were infected with JEV (5 MOI) in the presence of IFN-γ (10 ng/ml) for 48 h. The M1 polarization of infected BMDM was evaluated by intracellular IL-12p40 and iNOS staining combined with surface CD11b and F4/80 staining. **G** Higher production of M1 effector cytokines from NOX2-deificent macrophage by JEV infection. BMDM derived from WT and NOX2 KO mice was infected with JEV in the presence of IFN-γ, and the production of M1 effector cytokines (TNF-α, IL-6, IL-12p70) was determined by cytokine ELISA using culture media 48 h later. **H** Suppressed replication of JEV in NOX2-deficient macrophages. BMDM derived from WT and NOX2 KO mice were infected with JEV (1.0 MOI). Total RNA was extracted from infected BMDM and subjected to determine JEV replication with real-time qRT-PCR at the indicated time points post-infection. JEV RNA replication was expressed as viral RNA copy number targeted on JEV NS1 gene per microgram of total RNA. Data show the mean ± SEM of the levels derived from at least 2 individual experiments (n = 5–6). *p < 0.05, **p < 0.01, ***p < 0.001 between the levels derived from WT and NOX2 KO mice

Monocyte-derived M1 and M2 macrophages exhibit differential expression of surface markers. Consequently, M1 macrophages predominantly express high levels of CD80, CD86, MHC II, and TLR2, whereas CD206 and CXCR3 are highly expressed in M2 macrophages [55, 56]. Therefore, in order to further investigate the facilitated induction of M1 macrophage polarization due to NOX2 deficiency, the expression of surface molecules in M1 and M2 macrophages was analyzed. As anticipated, BMDM derived from NOX2 KO mice exhibited a significant increase in the expression of M1 macrophage surface markers, including CD80, CD86, MHC II, and TLR2, when compared to BMDM from WT mice (Fig. 5A). Conversely, the expression of M2 macrophage surface markers, CD206 and CXCR3, showed a slight decrease in BMDM derived from NOX2 KO mice. Furthermore, we examined the Ag-presenting capacity of BMDM derived from NOX2 KO mice, because the expression of molecules associated with Ag presentation (CD80, CD86, MHC II) was upregulated in M1-polarized macrophages [55, 56]. We conducted coculture experiments using CD4 ^+^ T cells isolated from OT-II Tg mice and BMDM derived from NOX2 KO and WT mice in the presence of OT-II CD4 ^+^ T cell epitope peptide (OVA_323–339_). Our results revealed that BMDM derived from NOX2 KO mice induced a stronger activation of OT-II CD4 ^+^ T cells. Notably, BMDM derived from NOX2 KO mice exhibited a significant increase of CD4 ^+^ T cells producing the autocrine growth factor IL-2 in the presence of OVA_323-339_ peptide when compared to those of WT mice (Fig. 5B). Similarly, BMDM derived from NOX2 KO mice induced increased production of IFN-γ from OT-II CD4 ^+^ T cells upon stimulation with OVA_323-339_ peptide, which indicates that NOX2-deficient BMDM facilitates Th1 differentiation of CD4 ^+^ T cells (Fig. 5C). When examining the expression of surface molecule indicatives of CD4 ^+^ T cell activation upon Ag presentation, BMDM derived from NOX2 KO mice were found to induce increased expression of CD44, CD154, as well as higher expression of CD25 and CD69, along with reduced expression of CD62L (Fig. 5D). This demonstrates that BMDM derived from NOX2 KO mice exhibit a stronger induction of OT-II CD4 ^+^ T cell activation compared to BMDM from WT mice. The primary ROS source initiates with the formation of a superoxide (O_2_^−^) and subsequently leads to the production of hydrogen peroxide (H_2_O_2_). This process is closely associated with NOX family, encompassing NOX1, NOX2, NOX3, NOX4, NOX5, DUOX1 and DUOX2 enzyme [57]. Thus, to ascertain the influence of H_2_O_2_ produced by NOX2 on de novo M1 and M2 macrophage polarization, we examined the effect of H_2_O_2_ addition on the de novo polarization of macrophages into M1 and M2 phenotypes. H_2_O_2_ addition in culture of BMDM derived from NOX2 KO mice markedly suppressed the production of M1 macrophage-related cytokines, IL-12p70, TNF-α, and IL-6 (Fig. 5E), which indicates that H_2_O_2_ produced by NOX2 could inhibit de novo polarization of macrophages into M1 phenotype. Furthermore, we investigated whether JEV infection could enhance M1 polarization of BMDM derived from NOX2 KO mice in the presence of IFN-γ. As a result, JEV infection was found to increase IL-12p40 and iNOS-producing macrophages in BMDM derived from NOX2 KO mice (Fig. 5F). Additionally, analysis of M1 macrophage-related cytokines, including IL-12p70, TNF-α, and IL-6, revealed that BMDM derived from NOX2 KO mice produced higher quantities of these cytokines in response to JEV infection (Fig. 5G). M1-polarized macrophages play a crucial role in inhibiting the replication of various viruses by producing antiviral cytokines and enhanced phagocytosis [33–36]. Therefore, we examined JEV replication in M1-polarized BMDM derived from NOX2 KO mice. As anticipated, it was observed that JEV replication in NOX2-deficient BMDM was significantly more inhibited than in BMDM derived from WT mice (Fig. 5H). Hence, these results suggest that macrophages derived from NOX2 KO mice undergo enhanced M1 macrophage polarization in response to JEV infection, thereby contributing to viral clearance at the early stage.

### Administration of ROS scavenger enhances resistance to JE

**Fig. 6:**
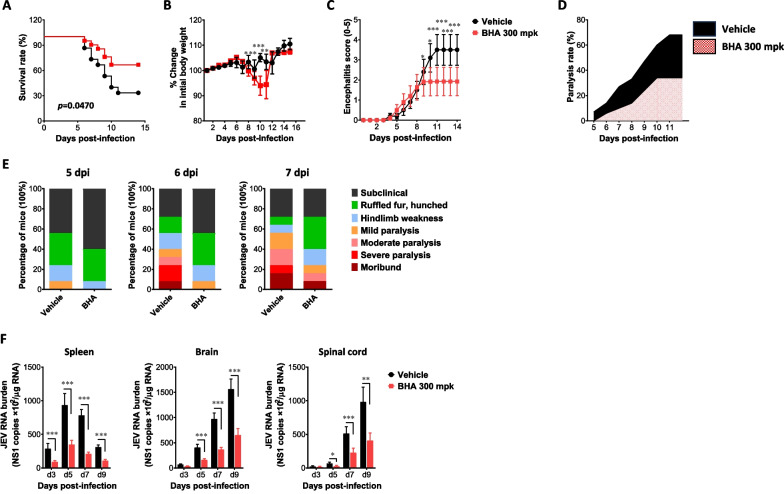
Administration of ROS scavenger attenuates JE progression. **A**–**C** Increased resistance of BHA-administered mice to JE. Wild-type (WT) C57BL/6 mice (5 to 6 weeks old, n = 10–11) were inoculated i.p. with JEV (7.5 × 10^7^ ffu), and daily oral administration of BHA 300 mpk was conducted from 5 days before infection to the 14th day of monitoring. **A**, Curve showing survival rate; **B**, Changes in body weight; **C**, Encephalitis score. **D** Ratio of BHA-administered mice showing neurological disorders during JE progression. BHA-administered WT mice were examined for neurological disorders every 6 h from 5 to 12 dpi. **E** Clinical signs. Clinical signs of BHA-administered WT mice were monitored and scored on day 5, 6, and 7 post-infection. **F** Viral burden in lymphoid and inflammatory tissues of BHA-administered mice during JE progression. Viral burden in lymphoid (spleen) and inflammatory tissues (brain and spinal cord) of BHA-administered WT mice (n = 5–6) were assessed by real-time qRT-PCR at the indicated time points after JEV infection. Viral RNA load was expressed as viral RNA copy number targeted on JEV NS1 gene per microgram of total RNA. Data show the mean ± SEM of the levels derived from at least 2 individual experiments (n = 5–6). *p < 0.05, **p < 0.01, ***p < 0.001 between the levels derived from WT mice administered with vehicle and BHA

Ultimately, our results suggest that ROS produced by NOX2 hinder M1 macrophage polarization, thereby preventing the proper induction of viral clearance in the peripheral tissues and potentially promoting JE progression. Butylated hydroxyanisole (BHA), known as a ROS scavenger, has been utilized in various diseases and health supplements with underscoring its potential utility in this context [58, 59]. Therefore, we became interested in whether BHA possesses inhibitory capabilities against JE progression caused by JEV infection. To assess the potential impact of BHA on the JE progression, we infected WT mice and orally administered BHA at a daily dose of 300 mpk. Subsequently, we observed changes in susceptibility to JE progression caused by JEV infection. Our results showed that BHA administration reduced susceptibility to JE progression caused by JEV infection. Specifically, WT mice administered with BHA showed approximately 30% mortality in response to JEV infection, while WT mice administered with a vehicle (corn oil) showed around 70% mortality (Fig. 6A). Furthermore, the body weight loss induced by JEV infection was less pronounced in WT mice treated with BHA (Fig. 6B), and symptoms of encephalitis were also less severe in BHA-treated mice (Fig. 6C). In support of this finding, BHA-treated WT mice showed reduced proportion of neurological disorders till 11 dpi, compared to vehicle-treated WT mice (Fig. 6D). Thus, this result indicate that BHA administration increases resistance to JE progression induced by JEV infection with reducing the manifestation of neurological symptoms. To clarify the effect of BHA on JE progression, we monitored BHA-treated WT mice for clinical signs depending on seven signs in the course of JE. Our results showed that BHA administered WT mice exhibited significantly reduced clinical signs from 5 to 7 dpi compared to vehicle-treated WT mice (Fig. 6E). To further examine the effect of BHA administration on JE progression, we assessed viral burden within lymphoid and the CNS tissues. As expected, BHA-administered WT mice contained less JEV burden in peripheral lymphoid tissue (spleen) and the CNS tissues (brain and spinal cord) (Fig. 6F). In particular, WT mice administered with BHA showed lower JEV detection in the peripheral lymphoid tissue during the early stage of infection and lower JEV detection in the CNS tissues later on. Therefore, these results paradoxically suggest that BHA administration as ROS scavenger inhibits JEV replication in the peripheral tissues, ultimately reducing the viral load invading the CNS, and lowering manifestation of acute encephalitis.

### ROS scavenger attenuates CNS inflammation by suppressing M1 polarization of macrophages in the course of JE

**Fig. 7:**
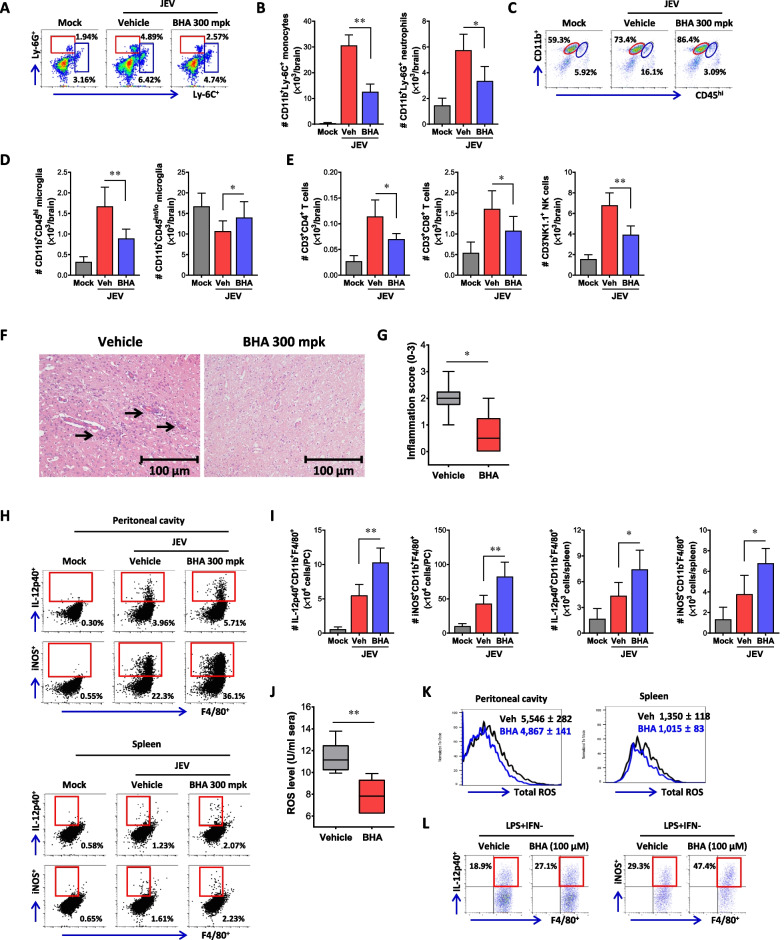
ROS scavenger attenuates CNS inflammation by suppressing M1 polarization of macrophages in the course of JE. **A** and **B** The frequency and number of Ly-6C^ +^ monocytes and Ly-6G ^+^ neutrophils in the brain of BHA-administered mice. Wild-type (WT) C57BL/6 mice (n = 5–6) were inoculated i.p. with JEV (7.5 × 10^7^ ffu), and BHA (300 mpk) was administered daily starting from 5 days before infection. The frequency (**A**) and total number (**B**) of Ly-6C ^+^ monocytes and Ly-6G ^+^ neutrophils in the brain were determined by flow cytometric analysis at 7 dpi using vigorous heart perfusion. Values in the dot-plots show the average percentage of each population after gating on CD11b^ +^ cells. **C** and **D** The frequency and number of activated and resting microglia in the brain of BHA-administered mice. The frequency (**C**) and total number (**D**) of CD11b^+^CD45^hi^ (activated microglia) and CD11b^+^CD45^int/lo^ (resting microglia) cells were determined by flow cytometric analysis at 7th dpi. **E** Total number of lymphoid cells in the brain. The total number of lymphoid cells (CD4 ^+^ and CD8 ^+^ T cells, NK cells) were assessed on the 7th dpi. **F** Histopathological examinations of the brain tissues derived from BHA-treated mice. Brain sections of vehicle and BHA-treated mice were prepared and stained with H&E 5 days after JEV infection via intraperitoneal route. Representative photomicrographs of the brain were obtained from blood vessel areas, meninges, and ventricles. **G** Inflammatory score of the brain tissues derived from BHA-administered mice. Inflammation was scored based on the degree of infiltration of inflammatory cells at 5th dpi. **H** Increased accumulation of M1 macrophages in peritoneal cavity and spleen of BHA-administered mice. **I** Accumulated number of M1 macrophages in the peritoneal cavity and spleen of BHA-administered mice. Leukocytes were prepared from the peritoneal cavity and spleen of BHA-administered WT mice 2 days after JEV infection, and briefly stimulated with LPS (200 ng/ml) for 6 h. M1 macrophages were then detected by intracellular IL-12p40 and iNOS staining combined with surface CD11b and F4/80 staining. **J** Reduced ROS levels in sera of BHA-administered mice. ROS levels were determined by ROS ELISA kit using sera derived from BHA-administered WT mice 2 days after JEV infection. **K** Intracellular ROS levels of CD11b ^+^ F4/80 ^+^ macrophages derived from BHA-administered mice. Intracellular ROS levels in macrophages derived from peritoneal cavity and spleen were assessed using total ROS assay kit 2 days after JEV infection. Values in histograms show the average MFI ± SEM of total ROS in macrophages population after gating on CD11b ^+^ F4/80 ^+^ cells. **L** Enhanced M1 polarization of BHA-treated macrophages. BMDM derived from BM cells of WT mice were stimulated with LPS and IFN-γ for 12 h in the presence of BHA (100 µM). The M1 polarization of BMDM was evaluated by intracellular IL-12p40 and iNOS staining combined with surface CD11b and F4/80 staining. Data show the mean ± SEM of the levels derived from at least 2 individual experiments (n = 5–6). *p < 0.05, **p < 0.01, ***p < 0.001 between the levels derived from WT mice administered with vehicle and BHA

To further characterize the CNS inflammation resulting from JEV infection in BHA-treated mice, we assessed the CNS infiltration of CD11b ^+^ Ly-6C ^+^ monocytes and CD11b ^+^ Ly-6G ^+^ neutrophils. Our results demonstrated the reduced frequency and number of infiltrated CD11b ^+^ Ly-6C ^+^ monocytes and CD11 ^+^ Ly-6G ^+^ neutrophils in the brain of BHA-administered WT mice compared to vehicle-administered WT mice (Fig. 7A and B). Notably, BHA-treated mice exhibited approximately three-fold reduction in the number of CD11b ^+^ Ly-6C ^+^ monocytes in the brain. Also, we assessed the frequency and number of resting and activated microglia cells in the brain of BHA-administered mice. Our results revealed a reduction in the frequency and number of CD11b ^+^ CD45^hi^ activated microglia with BHA administration, while the frequency and number of CD11b ^+^ CD45^int/lo^ resting microglia cells were modestly increased (Fig. 7C and D). Additionally, the number of infiltrated CD4 ^+^ , CD8 ^+^ T cells, and NK cells in the brain of BHA-administered mice was lower compared to vehicle-administered mice (Fig. 7E). These results indicate that BHA administration may mitigate neuroinflammation following JEV infection. To further assess the inhibition of CNS inflammation with BHA administration, we conducted histopathological examinations of the CNS tissues in BHA-treated WT mice. The results revealed less infiltration of inflammatory cells around brain blood vessels in BHA-administered mice compared to WT mice treated with a vehicle (Fig. 7F). Consistent with this observation, BHA-administered mice exhibited a decreased inflammation score in the brain compared to vehicle-administered mice, as determined by the extent of inflammatory cell infiltration (Fig. 7G). These findings suggest that the removal of ROS by BHA administration can inhibit CNS inflammation in JE progression following JEV infection. Next, similar to observations in NOX2 KO mice, we investigated whether BHA administration regulates M1 polarization of macrophages during JE progression following JEV infection. As expected, our results revealed that BHA-administered mice exhibited more enhanced accumulation of M1-polarized macrophages in the peritoneal cavity and spleen compared to vehicle-administered mice (Fig. 7H). Additionally, BHA-administered mice showed a higher number of accumulated IL-12p40 and iNOS-producing M1-polarized macrophages in the peritoneal cavity and spleen compared to vehicle-administered mice (Fig. 7I). Furthermore, BHA administration resulted in reduced ROS production in sera (Fig. 7J) and less production of total ROS in CD11b ^+^ F4/80 ^+^ macrophages derived from the peritoneal cavity and spleen (Fig. 7K). Finally, we examined whether BHA could regulate M1 polarization of BMDM derived from BM cells of WT mice. In the presence of BHA, BMDM exhibited increased induction of IL-12p40 and iNOS-producing M1 macrophages upon stimulation with LPS and IFN-γ (Fig. 7L). Overall, these results suggest that ROS removal with BHA administration is closely linked to the M1 polarization of macrophages, contributing to the regulation viral burden at the periphery and subsequently inducing less neuroinflammation following JEV infection.

## Discussion

The ROS produced by NOX is canonically recognized for their crucial role in the elimination of bacteria and viruses ingested by macrophages and neutrophils. Consequently, it has been thought that ROS generated by NOX inhibit viral infection and replication. NOX is a multisubunit enzyme complex comprising five crucial subunits, with two residing in the membrane and three in the cytosol [30]. ROS play a pervasive role in various aspect of cellular biology, exhibiting both detrimental and protective attributes [60–62]. Indeed, ROS produced by NOX induces type III IFN innate responses that result in providing antiviral effect against influenza A virus [60], while NOX2-derived ROS can regulate TLR7 on the cysteine residue Cys^98^ in negative feedback loop, thereby attenuating the antiviral responses [63]. Furthermore, dampening antiviral activity by ROS was reported to occur through oxidization of stimulator of interferon genes (STING) in herpesvirus infection [37]. Thus, the exacerbation of disease progression by ROS in viral infection appears to be indirectly induced through the oxidation of signaling molecules involved in type I/III innate responses, as well as tissue injury caused by ROS-mediated oxidation. However, there has been no research conducted to examine the influence of NOX2-derived ROS on immunopathological diseases resulting from viral infection by modulating macrophage polarization.

In this regard, the present study demonstrates that, contrary to an increased susceptibility, NOX2-deficient mice exhibit an augmented resistance to JE progression caused by JEV infection, as governed by the regulation of macrophage polarization. This finding ultimately signifies the contribution of ROS-mediated macrophage polarization to JE progression induced by JEV infection. Furthermore, our results revealed that NOX2 KO mice exhibited reduced viral load in peripheral lymphoid tissues and the CNS, accompanied by suppressed infiltration of inflammatory cells into the CNS, resulting in the attenuation of neuroinflammation. NOX2 KO mice showed enhanced JEV-specific Th1 CD4 ^+^ and CD8 ^+^ T cell responses compared to normal mice, along with increased accumulation of M1 macrophages producing IL-12p40 and iNOS in the peripheral lymphoid and inflamed tissues as extraneural tissues. Mechanistic experiments revealed that NOX2-deficient macrophages were found to differentiate into M1 phenotypes more prominently in response to viral infection, thereby leading to suppress viral replication. In particular, administration of H_2_O_2_ produced by NOX2 was shown to inhibit M1 macrophage polarization. Finally, the oral administration of ROS scavenger BHA increased resistance to JE progression induced by JEV infection and reduced viral load in the extraneural tissues as well as the CNS. Additionally, enhanced M1 polarization of macrophages with BHA administration strongly supports the notion that ROS removal facilitates M1 macrophage polarization, leading to suppressed viral replication in peripheral tissues. Therefore, our results imply that ROS generated by NOX2 contribute to viral replication within the peripheral extraneural tissues through the suppression of M1 macrophage polarization. Consequently, this leads to an augmentation in the viral load invading the CNS, ultimately facilitating JE progression.

NOX (NADPH oxidases) comprise a family of enzyme complexes with multiple isoforms primarily characterized by their catalytic subunit, “NOX” for membrane-spanning enzyme and “DUOX” for dual oxidase enzyme, facilitating the transfer of electrons from NADPH to molecular oxygen. Currently, seven members of the NOX family have been identified, encompassing NOX1 through NOX5, as well as DUOX1 and DUOX2-containing oxidase [30]. The predominant NOX isoform expressed in phagocytic cells, such as macrophages and neutrophils, is NOX2 oxidase. ROS production through NOX2 has been found to be increased in response to stimulation of pattern recognition receptors (PRRs), such as TLR7, following RNA virus infection like JEV [64]. In addition, NOX2 expressed in macrophages appears to play a crucial role in macrophage differentiation, as shown in previous study indicating that NOX2 deficiency suppressed M2 macrophage polarization along with diminishing ROS production [38]. However, this reveals a somewhat different phenomenon from our results, demonstrating that NOX2 deficiency leads to the inhibition of M1 macrophage polarization due to reduced ROS production. This difference may be attributed to variations in the characteristics of the macrophages utilized. Specifically, the previous research employed macrophage derived from BM monocytes by stimulation with M-CSF, thereby resulting in IRF4 expressing, M2 characterized macrophages, where NOX2 deficiency was shown to suppress M2 macrophage polarization in response to IL-4 treatment [38, 53, 54]. In contrast, our study used macrophages differentiated by GM-CSF, displaying an M1 characteristics [54], and showed enhanced M1 polarization in response to JEV infection and stimulation with LPS and IFN-γ, due to NOX2 ablation. Furthermore, our results indicate that NOX2-deficient macrophages displayed no changes in the expression of effector molecules for M2 polarized macrophages. Hence, it is plausible to infer that GM-CSF-derived macrophages used in this study manifest an M1-polarized phenotype characterized by IRF5 expression, thus indicating an augmented M1 polarization in response to JEV infection within the context of NOX2 deficiency.

Macrophage polarization has been proposed as a key factor in various immunopathological diseases. Macrophage activation occurs through stimulation by pathogen-associated molecular patterns (PAMPs), such as LPS and cytokines like IFN-γ, resulting in the differentiation of macrophages into the M1-polarized subtype [24–26]. In contrast, cytokines, such as IL-4 and IL-13, drive macrophage differentiation into the M2-polarized subtype. Recent studies unveiled additional subtypes within the M2-polarized macrophage category, such as M2a and M2b [65, 66]. The former M1 macrophages are well-known contributor to diverse inflammatory conditions, engaging in the elimination of invading bacteria and viruses, while also playing a role in inflammatory disorders, including autoimmune diseases [24–26]. In contrast, M2 macrophages possesses anti-inflammatory properties and are associated with tissue regeneration, although they have been implicated in disease progression, particularly in fibrosis induced by pulmonary diseases [65, 66]. Infections with flaviviruses like JEV have been shown to promote the differentiation of M1 macrophages, ultimately contributing to neuroinflammation in the brain [27, 28]. Furthermore, M1-polarized macrophages are known to enhance T cell responses by upregulating molecules involved in Ag presentation (CD80, CD86, MHC II), which can lead to increased CD4 ^+^ and CD8 ^+^ T cell activity [55, 56]. The CNS, including brain tissue, is considered an immunologically privileged tissue with finely regulated infiltration of inflammatory cells [67, 68]. The augmented accumulation of M1-polarized macrophages within brain tissue, particularly those derived from infiltrated Ly-6C ^+^ monocytes, has the potential to intensify neuroinflammation during JE progression [47]. However, NOX2 KO mice were found to show no significant difference in the distribution of M1-polarized macrophages producing IL-12p40 and iNOS in brain tissue, compared to wild-type normal mice (Data not shown). Therefore, the increased resistance observed in NOX2 KO mice during JE progression likely stems from the accumulation of M1-polarized macrophages in peripheral tissues. M1-polarized macrophages are highly effective in inhibiting viral replication and eliminating viruses by expressing various antiviral mechanisms, including increased phagocytic activity [26]. Hence, it can be inferred that the facilitated M1 macrophage polarization observed in NOX2 KO mice may effectively suppress viral replication in peripheral tissues when compared to normal mice. Furthermore, the heightened presence of M1-polarized macrophages in NOX2 KO mice is expected to induce robust IFN-γ and TNF-α-producing Th1 CD4 ^+^ and CD8 ^+^ T cell responses through their effective Ag presentation capabilities. In this context, it is noteworthy that BMDM derived from NOX2 KO mice exhibited enhanced Ag presentation capacity to OT-II CD4 ^+^ T cells. This result provides substantial support for our tentative hypothesis. Therefore, it is conceivable that the effective antiviral innate and adaptive immunity induced by the increased M1-polarized macrophages in NOX2 KO mice will play a substantial role in early restraining viral replication, notably in extraneural tissues, such as peripheral lymphoid tissues and sites of infection-related inflammation (peritoneal cavity). The reduction in viral load in the peripheral tissues of NOX2 KO mice will ultimately lead to a substantial decrease in the probability of viral load invading the CNS, thereby decreasing the onset of JE. This scenario is substantiated by the increased accumulation of M1-polarized macrophages and decreased viral load in peripheral tissues in NOX2 KO mice.

In this study, one of the most remarkable findings regarding the heightened resistance to JE attributed to facilitated M1 macrophage polarization in NOX2 KO mice is likely to be the outcomes obtained through the use of the ROS scavenger BHA. BHA is primarily employed as a food additive because of its antioxidative attributes, which serve to hinder or defer oxidation reactions and thereby prolong the shelf life of various products [69]. NOX2 facilitates the generation of superoxide O_2_^−^ by the transfer of electron to O_2_ from NADPH, and then conversion of O_2_^−^ to H_2_O_2_ by superoxide dismutase elicits various biological functions [70]. Synthetic phenolic compound BHA can scavenge ROS by providing a labile hydrogen atom to oxygen radical originating from fatty acids and other sources, thereby resulting in the formation of an oxidized phenolic ion that is stabilized by the intrinsic resonance of the benzene ring [69]. The extent and manner in which ROS influence the pro- and/or anti-inflammatory responses of macrophages (M1/M2 paradigm) depends on several factors, including the origin and localization of ROS and the specific stimulus applied [71]. However, research exploring ROS-mediated cellular signaling in macrophage activation and polarization is conspicuously limited. Although our study also did not delve into the intricacies of intracellular signaling pathways responsible for ROS-mediated inhibition of M1 macrophage polarization, the enhanced resistance to JE progression and reduced JEV load in peripheral tissues upon administration of the ROS scavenger BHA strongly substantiates the notion of ROS-mediated suppression of M1 macrophage polarization. In addition, the administration of NOX2-specific inhibitors like apocynin [72] also enhanced resistance to JE caused by JEV infection (Data not shown); however, it did not exhibit the same degree of efficacy as the ROS scavenger BHA. This discrepancy may perhaps be attributed to the speculation that ROS production occurs through different isoform of NOX [30, 64].

## Conclusions

Our findings suggest that inhibition of M1 macrophage polarization by ROS produced by NOX2 enzymatic actions contribute to the failure of controlling viral replication in peripheral tissues, leading to the progression of JE. These results underscore the potential significance of utilizing NOX2 inhibitor or ROS scavenger such as BHA for the critical management of fatal JE progression.

## Data Availability

The data supporting the conclusions of this article are included within the article. Original slides, photographs, and FACS dot-plots are retained. All reagents used in this study are available from scientific supply companies. The datasets used and analyzed during the current study are available from the corresponding author on reasonable request.

## Abbreviations

BBB: Blood–brain barrier
BHA: Butylated hydroxyanisole
BM: Bone marrow
CNS: Central nervous system
DCs: Dendritic cells
dpi: Days post-infection
IFN-I: Type I interferon
IFN-III: Type III interferon
JEV: Japanese encephalitis virus
KO: Knock-out
NOX2: NADPH oxidase 2
PAMPs: Pathogen-associated molecular patterns
PRRs: Pathogen recognition receptors
ROS: Reactive oxygen species
STING: Stimulator of interferon genes
WNV: West Nile virus
